# Expression of Interleukin-13 Receptor Alpha 2 in Brainstem Gliomas

**DOI:** 10.3390/cancers16010228

**Published:** 2024-01-03

**Authors:** Xiaoou Li, Xiong Xiao, Yi Wang, Guocan Gu, Tian Li, Yi Wang, Chunzhao Li, Peng Zhang, Nan Ji, Yang Zhang, Liwei Zhang

**Affiliations:** 1 Department of Neurosurgery, Beijing Tiantan Hospital, Capital Medical University, Beijing 100070, China; leexiaou@163.com (X.L.); neurocomputing@163.com (X.X.); wydeqingsu@163.com (Y.W.); gugc2018@163.com (G.G.); lt_ttyy@163.com (T.L.); wangyi19931027@sina.com (Y.W.); lichunzhao@ccmu.edu.cn (C.L.); zpengchina@126.com (P.Z.); jinan@bjtth.org (N.J.); 2 China National Clinical Research Center for Neurological Diseases, Beijing Tiantan Hospital, Capital Medical University, Beijing 100070, China; 3 Beijing Advanced Innovation Center for Big Data-Based Precision Medicine, Beihang University, Beijing 100191, China; 4 Beijing Neurosurgical Institute, Beijing Tiantan Hospital, Capital Medical University, Beijing 100070, China

**Keywords:** BSGs, brainstem gliomas, IL13Ra2, interleukin-13 receptor alpha 2, H3K27M, H3F3A

## Abstract

**Simple Summary:**

Brainstem gliomas, particularly those with the H3K27M mutation, represent highly aggressive tumors with limited therapeutic avenues. The exploration of tumor cell antigens in this context becomes pivotal to identifying potential treatment targets. Through multiplex immunofluorescence analysis, we unveiled a robust correlation between the widespread expression of the IL13Ra2 membrane antigen in brainstem glioma and the presence of the H3.3K27M antigen. This compelling association underscores the promise of IL13Ra2 as a prime therapeutic target for treating brainstem glioma.

**Abstract:**

The objective of this study was to investigate IL13Ra2 expression in brainstem glioma (BSG) and its correlation with key markers, functions, and prognostic implications, evaluating its therapeutic potential. A total of 80 tumor samples from BSG patients were analyzed. Multiplex immunofluorescence was used to examine six markers—IL13Ra2, H3.3K27M, CD133, Ki67, HLA-1, and CD4—establishing relationships between IL13Ra2 and these markers. Survival analysis, employing Kaplan–Meier and Cox proportional hazard regression models, encompassed 66 patients with complete follow-up. RNA-Seq data from a previously published study involving 98 patients were analyzed using the DESeq2 library to determine differential gene expression between groups. Gene Ontology (GO) enrichment and single-sample gene set enrichment analysis (ssGSEA) via the clusterProfiler library were used to delineate the gene functions of differentially expressed genes (DEGs). Nearly all the BSG patients displayed varying IL13Ra2 expression, with 45.0% (36/80) exhibiting over a 20% increase. Elevated IL13Ra2 levels were notably observed in pontine gliomas, diffuse intrinsic pontine gliomas (DIPGs), H3F3A-mutant gliomas, and WHO IV gliomas. IL13Ra2 expression was strongly correlated with H3.3K27M mutant protein, Ki67, and CD133. Patients with IL13Ra2 expression >20% showed shorter overall survival compared to those with ≤20% IL13Ra2 expression. The Cox proportional hazard regression model identified H3F3A mutations, rather than IL13Ra2 expression, as an independent prognostic factor. Analysis of RNA-Seq data from our prior cohort confirmed IL13Ra2’s correlation with H3.3, CD133, and Ki67 levels. Widespread IL13Ra2 expression in BSG, particularly elevated in the H3F3A mutant group, was strongly correlated with H3F3A mutations, increased proliferation, and heightened tumor stemness. IL13Ra2 represents a promising therapeutic target for BSGs, potentially benefiting patients with H3K27M mutations, DIPGs, WHO Grade IV, and pontine location-specific BSGs, particularly those with H3K27M mutations.

## 1. Introduction

Brainstem gliomas (BSGs) are a heterogenous group of gliomas that can present in the midbrain, pons, and medulla. BSGs exhibit distinct clinical characteristics and genetic features from cerebrum gliomas [1,2,3]. BSGs usually occur in children and account for 10% to 20% of all pediatric intracranial tumors, compared with 2% to 4% in adults [1,2,4]. Genetic mutations such as H3F3A (H3.3)-K27M and HIST1H3B/C (H3.1)-K27M are quite common and relatively unique for this type of glioma [5,6]. Given the densely concentrated vital neurofunctional structures within the brainstem, performing surgical resection for brainstem gliomas presents considerable challenges. With advances in surgical techniques and the widespread use of neuro-navigation and electrophysiological monitoring in the neurosurgical field, surgical resection has shown certain survival benefits for specific gliomas [7,8,9]. However, surgical risks and postoperative complications remain major concerns. The scope for safe surgical resection remains extremely limited for a considerable proportion of brainstem gliomas, particularly diffuse intrinsic pontine gliomas (DIPG), which account for 80% of pediatric cases [10]. Moreover, current treatments involving radiotherapy and chemotherapy with temozolomide only have minimal efficacy for the treatment of most BSGs, especially for BSGs with H3.3/H3.1-K27M mutations, for which no effective therapeutic option exists [11,12]. Thus, BSGs represent the most deadly brain cancer because the patients usually succumb to the disease less than 12 months after diagnosis [1,3]. Therefore, it is necessary to develop effective therapeutics for BSGs, especially those with H3.3/H3.1-K27M mutations.

Recent advances in T-cell immunotherapy and the prominent success of CAR-T-cell therapy in treating hematological malignancies have encouraged attempts to utilize these promising novel therapeutics in treating brain malignancies [13,14]. Despite the challenges encountered in CAR-T-cell immunotherapy for glioma, such as tumor heterogeneity, the tumor immunosuppressive microenvironment, and CAR-T-cell persistence, clinical trials have revealed significant potential for CAR-T-cell immunotherapy in treating glioma [15]. Some patients have experienced tumor shrinkage and an extended survival period [15,16]. Notably, a patient with CAR-T targeting the antigen IL13Ra2 achieved complete remission for up to 7.5 months, marking the first instance of CAR-T treatment achieving complete remission in a solid tumor [17]. Therapeutic agents labeled with IL-13 and other immunotherapies targeting IL13Ra2 have shown promise in the treatment of gliomas [18].

IL13Ra2 obstructs the activation of the STAT6 pathway by IL13RA1 and impedes normal cell apoptosis through its strong binding affinity with IL13, which is regarded as the primary carcinogenic mechanism of IL13Ra2. This mechanism is considered to have a close association with the initiation and progression of tumors [18,19,20]. Recent studies have uncovered its overexpression and implication in cancer development in a variety of malignancies, including pancreatic cancer, ovarian cancer, breast cancer, colon cancer, melanoma, and glioma [21], thus presenting a promising therapeutic target. In glioma, expression of IL13Ra2 is distinct among gliomas with different molecular subtypes and WHO malignancy grade and positively correlates with tumor proliferation and invasion and immune evasion abilities, leading to poorer patient outcomes [18,21,22,23,24]. 

Based on reports, IL13Ra2 is overexpressed at the protein and transcription levels in BSGs compared to normal brain tissue [25,26,27]. However, there has been inadequate reporting on its relationship with imaging and molecular subtypes, patient demographics, and microenvironment variables. Therefore, it remains to be determined whether IL13Ra2 is a potential target in BSGs, with these concerns yet to be addressed. 

Herein, we utilized two relatively large BSG cohorts to investigate IL13Ra2 expression at the protein and transcription levels and correlate it to clinical variables, molecular features, and malignant properties. Additionally, due to the correlation of IL13Ra2 found in other solid tumors and glioblastomas with tumor proliferation and immune cell infiltration [21,24,28], we examined the relationships between IL13Ra2 expression and the tumor stemness marker CD133, proliferation marker Ki67, important antigen presentation molecule HLA-1, helper T-cell marker CD4, and crucial brainstem glioma mutation H3K27M. This study aimed to investigate the potential of IL13Ra2 as a therapeutic target in brainstem gliomas.

## 2. Materials and Methods

### 2.1. Tumor Sample

A total of 80 patients with diagnosed pathological brainstem and thalamus gliomas were selected for this study (Table S1, Figure S1); 36 were tested for the H3F3A mutant gene. Their tumor tissues were collected at Beijing Tiantan Hospital, Capital Medical University, between 2008 and 2016 and stored in the hospital’s specimen bank. All the patients and their tumor samples fit the following criteria: (a) the tumor tissues were collected during their first surgical treatments or first biopsy procedures; (b) the patients had not underwent radiotherapies or chemotherapies before surgical treatments/biopsy and tissue collection; (c) their pathological diagnoses were confirmed and clear; (d) the tumor samples were well stored as formalin-fixed and paraffin-embedded (FFPE) samples; (e) the patients were followed up under the standardized follow-up protocol of the BSG group of BTH-CMU. After review by the neuropathologist, tumor samples that fit the following criteria were excluded from this study during selection: (a) necrosis percentage >50%; (b) total cellularity <50%; and (c) tumor nuclei <60%.

### 2.2. TMA Construction and Multiplex Immunofluorescence Analysis

TMAs (tumor microarrays) were constructed from FFPE tumor samples, including tissues from thalamic glioma (invading brain stem; *n* = 5), midbrain glioma (*n* = 14), pontine glioma (*n* = 38), and medulla oblongata glioma (*n* = 23). H&E staining was used to review the local pathology of the slides by a neuropathologist to ensure that the slides met the criteria (necrosis percentage ≤ 50%, total cellularity ≥ 50%, and tumor nuclei ≥ 60%).

Multiplex immunofluorescence staining (PerkinElmer, Waltham, MA, USA) was performed on FFPE slides for IL13Ra2 (1:400; #85677; CST, Danvers, MA, USA), H3.3 (1:800; #74829; CST, Danvers, MA, USA), Ki67 (1:400; ab70328; Abcam, Waltham, MA, USA), CD133 (1:400; #64326; CST, Danvers, MA, USA), CD4 (1:400; ab133616; Abcam, Waltham, MA, USA), HLA-1 (1:400; ab16667; Abcam, Waltham, MA, USA) and CD8 (1:200; ab4055; Abcam, Waltham, MA, USA). The secondary antibodies, dyes, and other reagents used in the experiments were sourced from the Opal 6-Plex Detection Kits (NEL811001KT, Akoya Biosciences, Marlborough, MA, USA). The whole slides were scanned, and multi-spectral images (MSI) were acquired at 20× for areas with tissue using the Vectra Polaris (PerkinElmer) multi-spectral imaging system. inForm 2.4.2 software (PerkinElmer) was used to remove autofluorescence, and for spectral unmixing and cell accounting. Firstly, standard multiplex fluorescence staining was performed on BSG-FFPE sections (without adding primary antibodies). Subsequently, these sections were scanned to capture the autofluorescent spectral features. Simultaneously, individual dyes corresponding to 6 channels between 520 and 690 were used for monochrome staining on typical positive tissues, which were then scanned to obtain the spectral features of these 6 channels. Single staining for each target protein was conducted to determine the optimal staining conditions, and then, each target was allocated to the appropriate channel to minimize crosstalk. Prior to the formal experiments, preliminary tests were conducted to confirm the optimal channel allocation for each target protein to reduce crosstalk. Following the completion of staining under the established optimal conditions from the preliminary tests, the resulting MSI files were spectrally unmixed using inForm software. During the unmixing process, the intrinsic fluorescence spectral features were used to shield the background, combined with the characteristic spectral features of each channel to perform the unmixing and evaluate the staining intensity in each channel.

### 2.3. Bioinformatic Confirmation and Analysis

Data from a previously published study from our group were included to provide bioinformatic confirmation of the findings. Ninety-eight patients from all the patients reported were selected as they had complete clinical variable and RNA-Seq data (Table S1, Figure S1) [29].

R (4.10.0) was used for the bioinformatic analyses. The relationships between IL13Ra2 expression and the clinical and molecular features of the tumors and oncological/immunological biomarkers were statistically tested. The IL13Ra2_group was classified according to the TPM value of the RNA-Seq results, and the patients were divided into IL13Ra2-low and IL13Ra2-high groups according to the median TPM value for IL13Ra2. The DESeq2 [30] library was used to analyze the groups’ differentially expressed genes (DEGs), and a heatmap was drawn to illustrate the distribution of the DEGs. GO enrichment [31] and ssGSEA [32] were performed using the clusterProfiler (4.6.2) library to uncover the gene functions of the DEGs.

The samples used in this study were obtained from patients who underwent surgery at Beijing Tiantan Hospital between 2008 and 2016. The RNA-Seq data used in this study was sourced from a previously published research study conducted between 2013 and 2017 [29]. Due to the earlier timeframe of sample collection, these pathological specimens lack the corresponding genetic mutation and molecular pathology data, making it challenging to classify them according to WHO CNS5 (World Health Organization Classification of Tumors of the Central Nervous System 5th Edition). Therefore, we have classified them according to WHO CNS4.

### 2.4. Western Blot

Cell lysates were prepared using RIPA lysis buffer (89901, ThermoFisher, Waltham, MA, USA) supplemented with a protease and phosphatase inhibitor cocktail (P1045, Beyotime, Shanghai, China). The extracted proteins were mixed with loading buffer (S6145-01, Scintol, Beijing, China) and boiled at 100 °C for 5 min. Subsequently, protein samples were subjected to electrophoresis on a NuPAGE Bis-Tris gel (4–12%, NP0335BOX, ThermoFisher, Waltham, MA, USA). Incubation with primary antibodies (H3.3K27M, 1:1000, #74829, CST, Danvers, MA, USA; IL13Ra2, 1:1000, #85677; CST, Danvers, MA, USA; CD133, 1:1000, ab19898, Abcam, Waltham, MA, USA; GAPDH, 1:3000, #97166, CST, Danvers, MA, USA) was conducted overnight at 4 °C, followed by incubation with secondary antibodies (anti-mouse IgG, #7076, CST, Danvers, MA, USA; anti-rabbit IgG, #7074s, CST, Danvers, MA, USA) for 2 h at room temperature. Chemiluminescence detection was performed using the SuperSignal West Pico Plus Chemiluminescent Substrate (34580, ThermoFisher, Waltham, MA, USA).

### 2.5. qPCR

RNA was extracted from the cell cultures using the SteadyPure Rapid RNA Extraction Kit (AG21023, Accurate Biology, Changsha, China). Subsequently, first-strand cDNA was synthesized using the S6 plus qPCR RT kit with gDNA remover (S6166-plus-01, Scintol, Beijing, China). Primer sets specific to IL13Ra2 (PRIM027787, BioTNT, Manassas, VA, USA), Ki67 (PRIM031093, BioTNT, Manassas, VA, USA), CD133 (PRIM037968, BioTNT, Manassas, VA, USA), and GAPDH (PRIM024970, BioTNT, Manassas, VA, USA) were employed. Quantitative PCR was performed on the Quant Studio 3 Real-Tme PCR system (ThermoFisher, Waltham, MA, USA).

### 2.6. Cell Culture

BSG cells were cultured in serum-free medium on plates coated with a matrigel matrix (1%, 37 °C, 4–12 h; 356234, Corning, Corning, NY, USA). The culture medium comprised DMEM (C11995500BT, Invitrogen, Carlsbad, CA, USA), B27 (17504044, Gibco), N2 (17502048, Gibco, Waltham, MA, USA), PDGF-AB (20 ng/mL; 100-00AB, PeproTech, Rocky Hill, NJ, USA), and 1% penicillin/streptomycin (03-033-1B, Biological Industries, Kibbutz Beit Haemek, Israel). The specific culturing procedures were consistent with those previously described. TT190313 is a H3K27M wild-type BSG cell. TT170720 and TT190326 are H3K27M mutant BSG cells.

### 2.7. Statistical Analysis

Based on a Kolmogorov–Smirnov test, the multidimensional fluorescence staining data exhibited a non-normal distribution. Therefore, these findings are presented as the median + interquartile range. On the other hand, after applying a logarithmic transformation, the RNA-Seq data display a normal distribution. Therefore, the associated results are presented as the mean + standard deviation. The Mann–Whitney U-test was employed for comparing two groups of nonparametric data, the *t*-test was used for comparing two groups of normally distributed data, and the Kruskal–Wallis test was used for comparing multiple groups of nonparametric data. Post hoc multiple comparisons were performed using the Dunn test following the Kruskal–Wallis analysis of variance. The relationships between the positive rate of IL13Ra2 cells and other markers were assessed using the Spearman correlation. The Wilcoxon matched-pair signed-rank test was used to assess the difference between positive cells and negative cells. The log-rank test was used to analyze the survival of patients. Statistical analysis was performed using Prism (9.5.1, GraphPad, La Jolla, CA, USA) and SPSS Statistics (26.0.0.0, IBM, Armonk, NY, USA), and *p*-values less than 0.05 were deemed statistically significant (ns *p* > 0.05, * *p* < 0.05, ** *p* < 0.01, *** *p* < 0.001, **** *p* < 0.0001).

## 3. Results

### 3.1. Brainstem Glioma Cohorts Included in This Study

IL13Ra2-targeting CAR-T therapy has shown encouraging results in treating supratentorial GBMs [18], which warrants further expansion of this therapy into other brain malignancies, such as BSGs. Therefore, comprehensively understanding the expression of its target, IL13Ra2, is a prerequisite for this expansion. In this study, we utilized two BSG cohorts (Table S1, Figure S1): one comprising 80 cases was used for the construction of tissue microarrays (TMAs); and another including 98 cases with available RNA-Seq data [29] for the bioinformatic analysis (BA). All the patients in both cohorts received neurosurgical resection to treat BSGs at Beijing Tiantan Hospital. The baseline characteristics of the TMA cohort are shown in Table S2. The TMA cohort included 39 adult (>14 years) and 41 pediatric (≤14 years) patients, comprising 5 cases in the thalamus (invading brain stem), 14 in the midbrain, 38 in the pons, and 33 in the medulla oblongata, according to the location that the main part of tumor occupies. There were 9 cases of grade I, 19 grade II, 3 grade III, and 49 grade IV gliomas, according to the WHO grading of tumors of the central nervous system. Moreover, 36 cases were diagnosed as DIPG; 36 had whole-exome sequencing data, and 22 harbored a K27M mutation in the gene H3F3A (H3F3A mutant). The BA cohort included 60 adult and 38 pediatric patients with a total of 54 pontine gliomas (31 were DIPGs), 30 medullary gliomas, and 14 midbrain gliomas. There were 7 grade I, 39 grade II, 31 grade III, and 21 grade IV gliomas. In the BA cohort, 56 tumors were H3F3A-mutant, while 40 tumors were H3F3A-wildtype. We utilized the TMA cohort to investigate the IL13Ra2 protein expression in BSGs, while the BA cohort was used to validate the TMA results at the transcriptional level and explore possible pathways that IL13Ra2 is involved in during the development of BSGs.

### 3.2. IL13Ra2 Staining in BSGs Tissues

**Figure 1:**
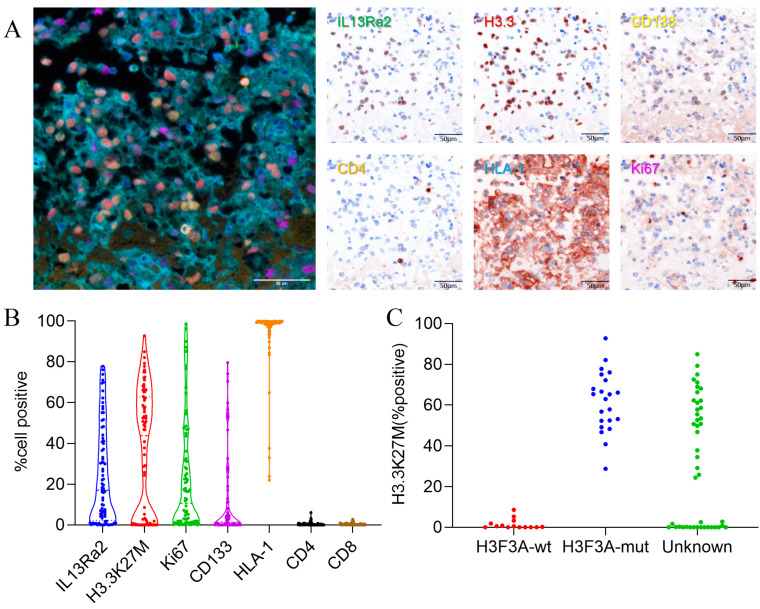
IL13Ra2 staining extent in BSG tissues. (**A**) Typical images of multiple fluorescence staining and spectral resolution of BSG FFPE tumor tissues (20×, all scale bars: 50μm); (**B**) expression of IL13Ra2, H3.3-K27M, Ki67, CD133, CD4, and HLA-1; (**C**) expression of H3.3K27M in H3F3A-mutant and wild-type tissues.

We used the method of multiplex immunofluorescence (MIF) staining to detect IL13Ra2 expression in the BSGs. This method allows the simultaneous staining of at most six protein markers. Therefore, we co-stained Ki67 (a cell proliferation marker), CD133 (a cancer stem cell marker), CD4 (representing CD4+ T-cell infiltration), HLA-I (human leukocyte antigen class I molecules), as well as H3.3-K27M (representing tumor cells harboring the H3F3A mutation), with IL13Ra2 in the same slide to further understand the expression pattern or features of IL13Ra2 in different types of BSGs (Figure 1A). We also conducted immunohistochemical, fluorescent staining for CD8 (a cytotoxic T-cell marker). The expression of these proteins was first automatically calculated and semi-quantified by inForm software. The expression levels were defined as staining extents by calculating the percentages of positively stained cells in one tissue core. As a result, the medians and ranges of the expression levels of IL13Ra2, H3.3-K27M, Ki67, CD133, HLA-1, CD4 and CD8 (median (IQR)) were 17.14% (2.28, 41.14), 43.37% (0.23, 62.78), 10.60% (1.16, 32.65), 1.34% (0.006, 11.27), 99.19% (97.09, 99.77), 0.16% (0.04, 0.47) and 0.20%(0.05, 0.47), respectively, in the whole TMA cohort (Figure 1B), reflecting a wide variety of expression of these targets among the different BSGs.

Notably, we compared the expression of H3.3-K27M between the H3F3A-mutant and wild-type BSG groups, which were categorized by the gene-sequencing method. We observed that all the wild-type tumors exhibited an H3.3-K27M expression of less than 10%, whereas the mutant tumors showed a much higher rate (Figure 1C). Therefore, we set an H3.3-K27M expression of 10% as the threshold to distinguish between the two groups. Thus, in the following analyses, samples that had not undergone gene sequencing for the H3.3-K27M mutation with a >10% H3.3-K27M expression in the tumor were considered H3.3 mutants, with the other samples classified as the wild-type group (Table S2).

### 3.3. High IL13Ra2 Expression Was Enriched in H3.3-Mutant BSGs

**Figure 2:**
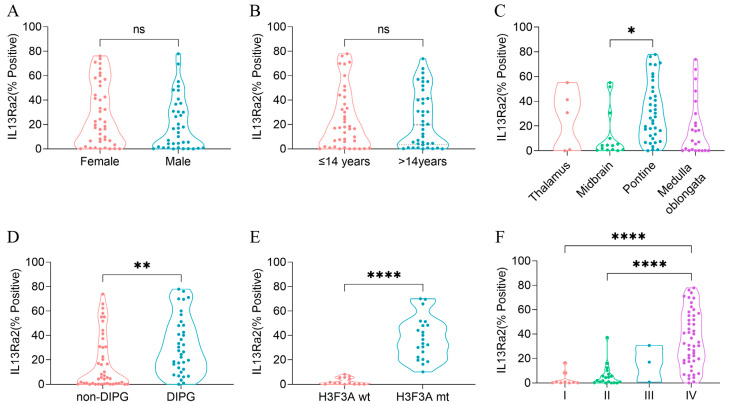
IL13Ra2 expression in different BSG subtypes of the TMA cohort. (**A**) IL13Ra2 expression of BSG tissues from patients of different genders; (**B**) IL13Ra2 expression of BSG tissues from patients of different ages; (**C**) expression of IL13Ra2 in different locations of BSG tumor tissues; (**D**) expression of IL13Ra2 in DIPG and non-DIPG tumor tissues; (**E**) expression of IL13Ra2 in H3F3A-wt and H3F3A-mt tumor tissues; (**F**) expression of IL13Ra2 in BSG tissues of different WHO grades (ns *p* > 0.05, * *p* < 0.05, ** *p* < 0.01, **** *p* < 0.0001).

To understand whether IL13Ra2 expression was substantially elevated in a specific type of BSGs, we compared the IL13Ra2 expression among different groups of BSGs (Figure 2). We did not observe a significant difference in expression between female and male populations (Figure 2A) or between adult (>14 years) and pediatric (≤14 years) patients (Figure 2B). However, a higher level of IL13Ra2 expression was observed in pontine gliomas (vs. midbrain gliomas, *p* = 0.0099, Figure 2C), DIPGs (vs. non-DIPG, *p* = 0.0013, Figure 2D), H3F3A-mutant gliomas (vs. the H3F3A wild-type, *p* < 0.0001, Figure 2E), as well as WHO grade IV gliomas (vs. grade I or II gliomas, *p* < 0.0001, Figure 2F), indicating distinct IL13Ra2 expression patterns among different BSGs. 

Next, we subtyped the BSGs of the TMA cohort into different IL13Ra2 expression groups according to different thresholds (Table S2). As a result, the high-IL13Ra2 expression groups were consistently enriched in the WHO grade IV and H3.3-mutant BSGs, indicating that the WHO grades and H3F3A mutation are the two factors profoundly impacting IL13Ra2 expression. Of note, a large fraction of H3.3-mutant BSGs expressed an extremely high level of IL13Ra2, such as a >50% expression of IL13Ra2 in approximately 32% (15/47) of H3.3-mutant cases (Table S2), suggesting that this BSG type would be a suitable target for IL13Ra2-targeted CAR-T therapies.

Furthermore, we constructed a fitted multiple linear regression model that includes gender, age, and the aforementioned variables related to IL13Ra2 expression. As a result, the H3.3 mutant emerged as the only factor independently affecting IL13Ra2 expression in BSGs (Table S3), indicating that the H3.3 mutation is the most significant factor impacting IL13Ra2 expression. 

### 3.4. IL13Ra2 Expression Was Enriched in Glioma Cells Harboring the H3F3A Mutation, Proliferation Features, and Stem Cell Properties

**Figure 3:**
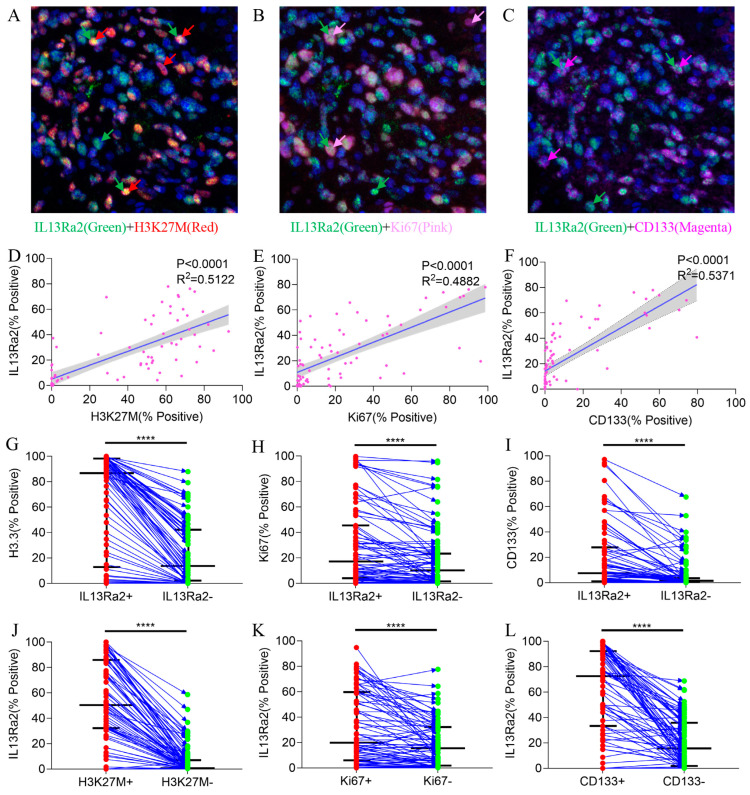
Co-staining of IL13Ra2 with H3.3-K27M, Ki67, and CD133. Images showing co-expression of IL13Ra2 and H3K27M (**A**), Ki67 (**B**), and CD133 (**C**) in BSG tumor tissues. The linear regression results between IL13Ra2 expression and H3K27M (**D**), Ki67 (**E**), and CD133 (**F**) expression in BSG tumor tissue. Comparison of the positive rates of H3K27M (**G**), Ki67 (**H**), and CD133 (**I**) in IL13Ra2-positive and negative cells from different tumor samples. Comparison of the positive rates of IL13Ra2 in H3K27M-positive/H3K27M-negative (**J**), Ki67-positive/Ki67-negative (**K**), and CD133-positive/CD133-negative (**L**) tumor cells across different tumor tissues (**** *p* < 0.0001).

Next, we investigated the features of BSG cells expressing IL13Ra2 since the MSI method allows for simultaneous staining of IL13Ra2 with other markers in the same slide (Figure 3A–C). Analyses of the Spearman correlations between the expression levels of IL13Ra2 and those of H3.3K27M, CD133, and Ki67 revealed robust associations (Table S4). IL13Ra2 expression was highly correlated with the expression of H3.3K27M-mutant protein (*p* < 0.0001, R^2^ = 0.5122, Figure 3D), Ki67 (a cell proliferation marker; *p* < 0.0001, R^2^ = 0.4882, Figure 3E), and CD133 (a stem cell marker; *p* < 0.0001, R^2^ = 0.5371, Figure 3F). Furthermore, the IL13Ra2-expressing cells had a higher proportion of H3.3K27M, Ki67, and CD133 expression than cells that did not express IL13Ra2 (Figure 3G–I). Moreover, the proportion of IL13Ra2 expression in cells expressing H3K27M, Ki67, and CD133 was higher than that in cells not expressing H3K27M, Ki67, and CD133, respectively (Figure 3J–L). We conducted Western blot experiments using tissue samples from three cases of H3K27M mutant-type BSG tumors and three cases of H3K27M wild-type BSG tumors. The results demonstrated higher expression levels of IL13Ra2 and CD133 in the H3K27M mutant-type tumors, consistent with the aforementioned experimental trends (Figure S2E). Additionally, we performed Western blot experiments using available BSG cells, which similarly indicated elevated expression levels of IL13Ra2 and CD133 in H3K27M mutant-type tumor cells (Figure S2G). All these findings indicate that IL13Ra2 expression is enriched in cell populations that harbor the H3K27M mutation, proliferation features, or stem cell properties (Figure 3). Since these cells represent a highly malignant group of glioma cells in BSGs, CAR-T therapies targeting these cells would facilitate better control of tumor growth, leading to a survival benefit over using other targets. However, there was no significant correlation between IL13Ra2 expression and CD4/8 T-cell density (Figure S2A,B) or HLA-1 expression (Figure S2C), suggesting no impact of IL13Ra2 expression on T-cell infiltration or antigen presentation.

### 3.5. Higher IL13Ra2 Expression Associated with Shorten Overall Survival 

**Figure 4:**
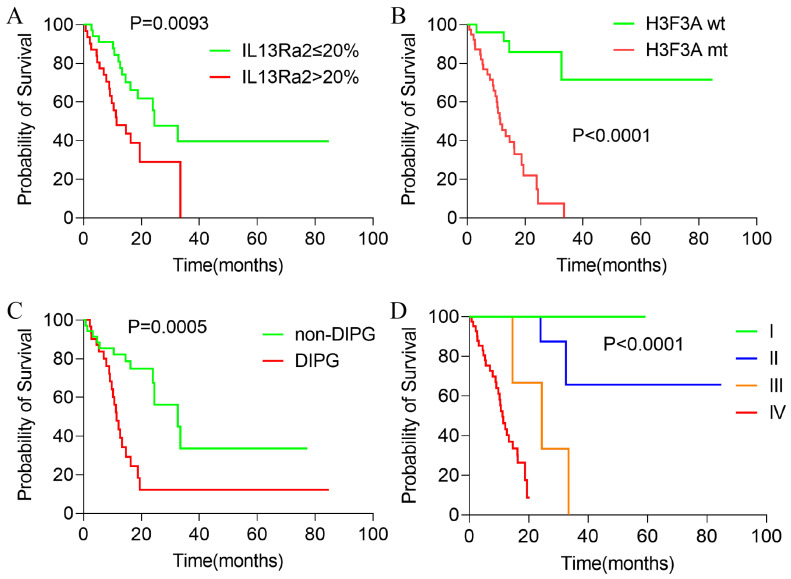
Kaplan–Meier survival analysis of patients with BSGs in the TMA cohort. Kaplan–Meier survival analysis results of BSG patients with different IL13Ra2 expressions (**A**), different H3K27M mutation statuses (**B**), DIPG and non-DIPG types (**C**), and different WHO grades (**D**).

Since IL13Ra2 expression was correlated with malignant phenotypes in BSGs, we asked whether IL13Ra2 expression affects the outcomes of BSG patients. Among the 80 cases in the TMA cohort, 66 cases had full follow-up information. The median overall survival was 19.48 months after a median follow-up time of 20.63 months. As was expected and consistent with previous reports, H3F3A mutation, DIPG appearance, and WHO malignancy grade significantly impacted the patient outcomes (Figure 4A–C). Cases with an IL13Ra2 expression >20% exhibited shorter overall survival times compared with the cases with a ≤20% IL13Ra2 expression (11.40 months vs. 24.47 months, *p* = 0.0095, Figure 4D). However, no significant difference was observed between the two groups stratified by other thresholds of IL13Ra2 expression. Moreover, a Cox proportional hazard regression model that included age, gender, presence of H3F3A mutation, DIPG appearance, WHO malignancy grade, and >20% IL13Ra2 expression showed that the H3K27 mutation, not the IL13Ra2 expression, was an independent prognosticator in the TMA cohort (Table S5). These findings indicate that the association of the >20% IL13Ra2 expression with unfavorable outcomes relies on the enrichment of H3K27M-mutant cases in this group of patients. 

### 3.6. IL13Ra2 and Pathways Promoting BSG Progression

**Figure 5:**
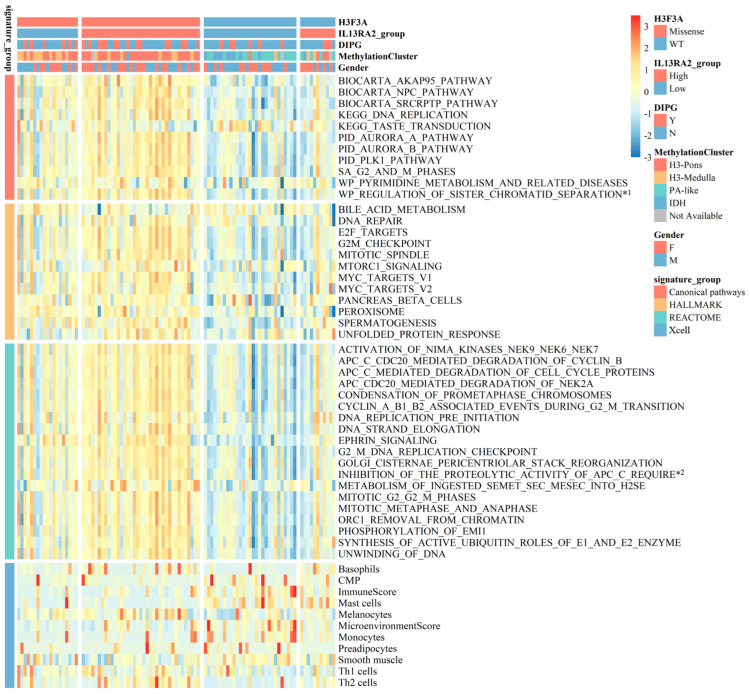
Heatmap of the BA cohort. A heatmap shows the differences in functional pathways and cellular components between the IL13Ra2-high group and IL13Ra2-low group of tumor tissues of the BA cohort. *1: WP_REGULATION_OF_SISTER_CHROMATID_SEPARATION_AT_THE_METAPHASEANAPHASE_TRANSITION; *2: INHIBITION_OF_THE_PROTEOLYTIC_ACTIVITY_OF_APC_C_REQUIRED_FOR_THE_ONSET_OF_ANAPHASE_BY_MITOTIC_SPINDLE_CHECKPOINT_COMPONENTS.

To explore the pathways that would mediate the link between IL13Ra2 expression and BSG malignancy, we performed single-sample gene set enrichment analyses (ssGSEA) on the RNA-Seq data from the BA cohort [29], and examined the differences in molecular pathway activity between BSGs with high and low IL13RA2 expression. A total of 10 Hallmark and 28 Reactome gene sets emerged as molecular pathways that showed enhanced activity in the IL13Ra2-high group (Figure 5). A wide variety of molecular functions, including cell proliferation, cell metabolism, and DNA repair, were involved, suggesting an extensive impact of IL13Ra2 on BSG malignancy. Meanwhile, tumors with lower immune scores and microenvironment scores were enriched in the IL13Ra2-high group, indicating a possible negative impact of IL13Ra2 on immune infiltration (Figure 5).

**Figure 6:**
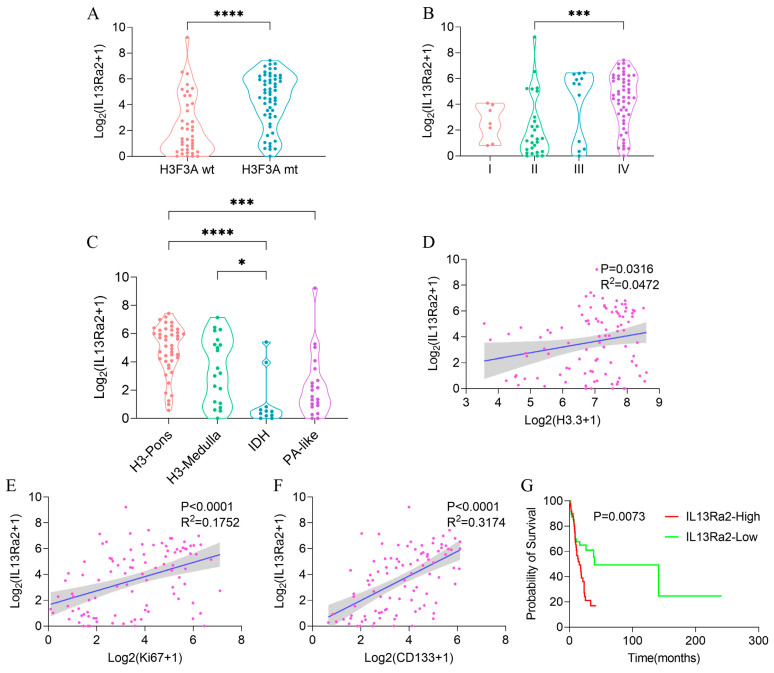
IL13Ra2 expression in BSGs of the BA cohort. Comparison of IL13Ra2 transcription level in tumor tissues with different H3F3A statuses (**A**), WHO grades (**B**), and methylation subtypes (**C**) in the BA cohort. The linear regression analysis results for IL13Ra2 transcription level and H3.3 (**D**), Ki67 (**E**), and CD133 (**F**) transcription levels in the BA cohort. (**G**) Kaplan–Meier survival analysis results of the IL13Ra2-high group and IL13Ra2-low group in the BA cohort (* *p* < 0.05, *** *p* < 0.001, **** *p* < 0.0001).

Consistent with the TMA results, we observed a higher transcriptional level of IL13Ra2 in cases with the H3.3-K27M mutation (Figure 6A, *p* < 0.0001) or grade IV malignancy (Figure 6B, *p* = 0.0007), and also significant correlations of the transcriptional levels of IL13Ra2 with H3.3-K27M (*p* < 0.0316 R^2^ = 0.0472, Figure 6D), Ki67 (*p* < 0.0001 R^2^ = 0.1752, Figure 6E), and CD133 (*p* < 0.0001 R^2^ = 0.3174, Figure 6F) transcriptional levels. We conducted qPCR experiments using tissue samples from three cases of H3K27M mutant-type BSG tumors and three cases of H3K27M wild-type BSG tumors. The results revealed higher relative expression levels of IL13Ra2, Ki67, and CD133 in the H3K27M tumor tissues (Figure S2D). Subsequently, qPCR experiments using available BSG cells demonstrated a trend consistent with the qPCR results obtained for the tumor tissues (Figure S2F). Moreover, the H3-pons subtype, in which the H3.3-K27M mutation is enriched [29], exhibited the highest transcriptional level of IL13Ra2 among the four methylation subtypes (Figure 6C, *p* < 0.0001). Finally, the IL13Ra2-high group also showed shorter survival times than the low group (Figure 6G, *p* = 0.0073). This link is also attributed to the enrichment of the H3.3-K27M mutation, as suggested by the multivariate Cox regression (Table S6) that did not identify IL13Ra2 expression as an independent risk factor for survival. Therefore, all the findings from the RNA-Seq data suggest that IL13Ra2 expression is associated with increased malignancy, such as high cell proliferation, cancer stem cell properties, and malicious genetic mutations (H3.3-K27M), which were also revealed in the TMA study.

## 4. Discussion

BSGs are a group of gliomas with clinical characteristics and genetic features that are distinct from supratentorial gliomas that can present in the cerebrum [29]. A significant portion of brainstem gliomas (BSGs) carry the H3.3-K27M mutation, classifying them as diffuse midline gliomas (DMGs) under both the 2016 and 2021 versions of the WHO Classification of Tumors of the Central Nervous System [29,33,34]. This disease entity represents the most refractory brain cancer, with a universally abysmal survival of approximately 10 months [1,2,4] due to its inaccessible location for surgical resection as well as full resistance to radiation and chemotherapy. 

Thus, new therapeutics are urgently needed. IL13Ra2-targeting CAR-T-cell immunotherapy has generated an encouraging result in treating supratentorial GBMs [17,18], stirring intensive interest in expanding the therapy to BSGs. There are six ongoing clinical trials, registered in clinicaltrial.gov, exploring its safety and efficacy in treating a variety of brain malignancies, including melanoma, ependymoma, medulloblastoma, and glioblastoma. Recent reports highlight the promising effects of novel immune cell therapies targeting IL13Ra2, such as bispecific T-cell engagers (BiTEs) and bispecific killer cell engagers (BiKEs), demonstrating their cytotoxicity against GBM, potentially showing more therapeutic potential than traditional immune cell therapies [35,36]. Apart from immune cell therapies, targeting IL13Ra2 with antibody treatments is another promising avenue. Antibodies targeting IL13Ra2 offer higher specificity compared to natural IL13 ligands, effectively penetrating the blood–brain barrier and homing into tumors, showing considerable therapeutic potential [37,38]. Dendritic cells sensitized with glioma-associated antigens (GAAs) represent a notable direction in immunotherapy research. Studies demonstrate that dendritic cell therapy results in sensitization to IL13Ra2 antigens, induces an immune response, and results in a certain level of glioma cell cytotoxicity [39,40,41]. Clinical trials of peptide-pulsed dendritic cell vaccines targeting multiple peptides (including IL13Ra2) exhibit good safety profiles, immunoreactivity, and potential efficacy [42,43]. In addition to immunotherapy, targeted drug delivery that targets IL13Ra2 has shown promise. Early attempts with chimeric proteins consisting of IL13 ligands and Pseudomonas exotoxins demonstrate cytotoxicity against glioma cells. Strategies combining diphtheria toxin with IL13 ligands also exhibit potential therapeutic efficacy [44,45]. Subsequent methods using extracellular toxins specifically targeting IL13Ra2 show certain anti-glioma activities with enhanced specificity and minimal neurotoxicity. Coupled with convection-enhanced delivery (CED), their safety and effectiveness are further reinforced [46,47,48]. Viral vectors expressing IL13 ligands have been developed as potential gene therapy carriers, effectively transferring adenoviruses and lentiviruses into glioma cells [49,50]. Oncolytic measles virus strains, exhibiting anti-glioma activity after IL-13 modification, demonstrate significant cytotoxicity with enhanced specificity [51].

Liposomes, nano-sized artificial vesicles that can be loaded with drugs, when modified with IL-13, effectively deliver drugs to malignant glioma cells, showing superior efficacy compared to non-targeted delivery [52,53]. Combining certain inorganic nanoparticles with IL13Ra2 antibodies demonstrates targeted anti-glioma activity against glioma cells; such strategies include high-performance nano-biocatalysts and biocompatible magnetic vortex discs [54,55]. Additionally, recent studies have identified that protein tyrosine phosphatase-1B inhibitors inhibit IL13 signal transduction, attenuating the invasive and migratory capabilities of IL13Rα2-positive GBM tumor cells, showing promising therapeutic potential in animal experiments [56]. While these techniques are still in their exploratory stages, the potential value of targeting IL13Ra2 for glioma therapy is undeniable. 

Mounting evidence has shown an overexpression of IL13Ra2 in a fraction of supratentorial gliomas, especially in GBMs [18,21]. However, studies on whether and to what extent IL13Ra2 protein is expressed in BSGs are still lacking. Moreover, the clinical characteristics, genetic features, and malignant phenotypes of cases with high IL13Ra2 expression remain unknown. A comprehensive understanding of IL13Ra2 expression in BSG tumor samples is paramount since it will facilitate the development and improvement of immunotherapy against this deadly brain cancer. 

In this study, we utilized two relatively large BSG cohorts, comprising 80 and 98 BSG cases, to investigate IL13Ra2 expression at the protein and transcriptional levels, respectively. We observed that IL13Ra2 was expressed in a large fraction of BSGs (Figure 1 and Table S2), especially in those with highly malignant molecular features or phenotypes, such as the presence of the H3.3-K27M mutation, an elevated WHO grade, increased cell proliferation, and enhanced stem cell properties (Figure 2 and Figure 3). All these findings indicate that IL13Ra2 is a good target for CAR-T-cell therapy and other therapeutics since it leads to the specific killing of cancer cells that are the most resistant to current therapeutics and, thus, the origin of tumor recurrence [57]. 

We also explored the relationship between IL13Ra2 expression and some immune features (Figure 5 and Figure S2A–C). Although we did not observe any correlations of IL13Ra2 expression with CD4/8 T-cell infiltration or HLA-1 expression, we noticed the HLA-1 expression was not suppressed in the majority of BSG tumor samples. This finding suggests that antigen presentation via the MHC-1 route may be intact in BSGs, compared with other malignancies such as melanoma, colorectal, bladder, head and neck, breast, lung, kidney, prostate, and cervical carcinoma [58], in which this route is usually suppressed. Thus, immunotherapies that rely on the intact function of the MHC-1 route, such as tumor vaccine and TCR (T-cell receptor)–T-cell therapy, would, in theory, be effective for this fatal cancer. While we did not observe a correlation between IL13Ra2 expression and T-cell infiltration, RNA-Seq data revealed that the group with high IL13Ra2 expression exhibited lower immune and microenvironment scores. Recent research has highlighted the potential role of glioma-associated macrophages in glioblastoma progression and the formation of an immunosuppressive milieu within glioblastomas [59,60]. Likewise, prior studies have identified macrophages as crucial elements in shaping the non-inflammatory tumor environment in DIPG [61]. 

Exploring the relationship between IL13Ra2 and macrophage functional status represents a promising avenue for further investigation. In addition, the H3.3-K27M mutation has proven to be a promising target for these immunotherapies in pre-clinical studies [62], and a few early-phase trials are investigating their safety and efficacy in treating BSGs [63]. Interestingly, we observed a high correlation between the expression of IL13Ra2 and H3.3-K27M at both the tissue (Figure 3D) and cell (Figure 3A,D,J) levels, suggesting a potentially synergetic effect between immunotherapies targeting IL13Ra2 and the H3.3-K27M mutation. 

This study also has several limitations. Firstly, due to the low overall incidence and limited population suitable for the surgical treatment of brainstem gliomas, coupled with challenges in specimen acquisition, the total number of cases included is relatively small. However, within the specific field of brainstem glioma research, this study qualifies as having a large sample size. Secondly, due to the extended time span of the sample collection and difficulties in acquiring specimens, this study was unable to simultaneously obtain samples suitable for multiplex immunofluorescence staining and matched RNA-Seq data from the same set of specimens. For similar reasons, the samples and RNA-Seq data utilized in this study originated from patients treated at Beijing Tiantan Hospital between 2008 and 2017, thereby classified using the 2016 WHO CNS4 classification. As a result of the earlier sample collection period, these pathological specimens lack corresponding genetic mutations and molecular pathology data, making it challenging to classify them according to WHO CNS5 criteria. Despite these limitations, our study sufficiently demonstrates the widespread expression of IL13Ra2 in brainstem gliomas and its close association with H3K27M, suggesting its potential as a therapeutic target for brainstem gliomas.

## 5. Conclusions

IL13Ra2 is overexpressed in a large proportion of BSGs, especially in tumors harboring the H3.3-K27M mutation, undergoing cell proliferation, or possessing stem cell properties. Despite the challenges in various targeted therapies, ongoing research continues to advance, highlighting IL13Ra2 as a promising therapeutic target for brainstem gliomas.

## Data Availability

The data presented in this study are available on request from the corresponding author.

## Supplementary Materials

The following supporting information can be downloaded at https://www.mdpi.com/article/10.3390/cancers16010228/s1. Figure S1: The workflow of this study; Figure S2: Co-staining results in the TMA cohort and Western blot and qPCR results of BSG tumor tissue and cells; Table S1: Clinical characteristics of TMA cohort and BA cohort; Table S2: IL13Ra2 expression in different BSG subgroups in TMA cohort; Table S3: Multiple linear regression of IL13Ra2 expression in TMA cohort; Table S4: Spearman correlation analyses of IL13Ra2 expression in TMA cohort; Table S5: Multivariate Cox regression for survival analysis in TMA cohort; Table S6: Multivariate Cox regression for survival analysis in BA cohort.

## References

[1] Grimm S.A., Chamberlain M.C. (2013). Brainstem glioma: A review. Curr. Neurol. Neurosci. Rep..

[2] Hu J., Western S., Kesari S. (2016). Brainstem glioma in adults. Front. Oncol..

[3] Wummer B., Woodworth D., Flores C. (2021). Brain stem gliomas and current landscape. J. Neuro-Oncol..

[4] Ostrom Q.T., Gittleman H., Fulop J., Liu M., Blanda R., Kromer C., Wolinsky Y., Kruchko C., Barnholtz-Sloan J.S. (2015). Cbtrus statistical report: Primary brain and central nervous system tumors diagnosed in the united states in 2008-2012. Neuro-Oncology.

[5] Khuong-Quang D., Buczkowicz P., Rakopoulos P., Liu X., Fontebasso A.M., Bouffet E., Bartels U., Albrecht S., Schwartzentruber J., Letourneau L. (2012). K27m mutation in histone h3.3 defines clinically and biologically distinct subgroups of pediatric diffuse intrinsic pontine gliomas. Acta Neuropathol..

[6] Schwartzentruber J., Korshunov A., Liu X., Jones D.T.W., Pfaff E., Jacob K., Sturm D., Fontebasso A.M., Quang D.K., Tönjes M. (2012). Driver mutations in histone h3.3 and chromatin remodelling genes in paediatric glioblastoma. Nature.

[7] Ius T., Lombardi G., Baiano C., Berardinelli J., Romano A., Montemurro N., Cavallo L.M., Pasqualetti F., Feletti A. (2023). Surgical management of adult brainstem gliomas: A systematic review and meta-analysis. Curr. Oncol..

[8] Zhang M., Xiao X., Gu G., Zhang P., Wu W., Wang Y., Pan C., Wang L., Li H., Wu Z. (2023). Role of neuronavigation in the surgical management of brainstem gliomas. Front. Oncol..

[9] Pan C., Zhang M., Xiao X., Kong L., Wu Y., Zhao X., Sun T., Zhang P., Geng Y., Zuo P. (2023). A multimodal imaging-based classification for pediatric diffuse intrinsic pontine gliomas. Neurosurg. Rev..

[10] Rashed W.M., Maher E., Adel M., Saber O., Zaghloul M.S. (2019). Pediatric diffuse intrinsic pontine glioma: Where do we stand?. Cancer Metastasis Rev..

[11] Gwak H.S., Park H.J. (2017). Developing chemotherapy for diffuse pontine intrinsic gliomas (dipg). Crit. Rev. Oncol./Hematol..

[12] Johung T.B., Monje M. (2017). Diffuse intrinsic pontine glioma: New pathophysiological insights and emerging therapeutic targets. Curr. Neuropharmacol..

[13] Grosser R., Cherkassky L., Chintala N., Adusumilli P.S. (2019). Combination immunotherapy with car t cells and checkpoint blockade for the treatment of solid tumors. Cancer Cell.

[14] Singh A.K., Mcguirk J.P. (2020). Car t cells: Continuation in a revolution of immunotherapy. Lancet Oncol..

[15] Zhang P., Zhang Y., Ji N. (2022). Challenges in the treatment of glioblastoma by chimeric antigen receptor t-cell immunotherapy and possible solutions. Front. Immunol..

[16] Akhavan D., Alizadeh D., Wang D., Weist M.R., Shepphird J.K., Brown C.E. (2019). Car t cells for brain tumors: Lessons learned and road ahead. Immunol. Rev..

[17] Brown C.E., Alizadeh D., Starr R., Weng L., Wagner J.R., Naranjo A., Ostberg J.R., Blanchard M.S., Kilpatrick J., Simpson J. (2016). Regression of glioblastoma after chimeric antigen receptor t-cell therapy. N. Engl. J. Med..

[18] Thaci B., Brown C.E., Binello E., Werbaneth K., Sampath P., Sengupta S. (2014). Significance of interleukin-13 receptor alpha 2-targeted glioblastoma therapy. Neuro-Oncology.

[19] Rahaman S.O., Sharma P., Harbor P.C., Aman M.J., Vogelbaum M.A., Haque S.J. (2002). Il-13r(alpha)2, a decoy receptor for il-13 acts as an inhibitor of il-4-dependent signal transduction in glioblastoma cells. Cancer Res..

[20] Arima K., Sato K., Tanaka G., Kanaji S., Terada T., Honjo E., Kuroki R., Matsuo Y., Izuhara K. (2005). Characterization of the interaction between interleukin-13 and interleukin-13 receptors. J. Biol. Chem..

[21] Zeng J., Zhang J., Yang Y.Z., Wang F., Jiang H., Chen H.D., Wu H.Y., Sai K., Hu W.M. (2020). Il13ra2 is overexpressed in malignant gliomas and related to clinical outcome of patients. Am. J. Transl. Res..

[22] Brown C.E., Warden C.D., Starr R., Deng X., Badie B., Yuan Y., Forman S.J., Barish M.E. (2013). Glioma il13rα2 is associated with mesenchymal signature gene expression and poor patient prognosis. PLoS ONE.

[23] Bhardwaj R., Suzuki A., Leland P., Joshi B.H., Puri R.K. (2018). Identification of a novel role of il-13rα2 in human glioblastoma multiforme: Interleukin-13 mediates signal transduction through ap-1 pathway. J. Transl. Med..

[24] Han J., Puri R.K. (2018). Analysis of the cancer genome atlas (tcga) database identifies an inverse relationship between interleukin-13 receptor α1 and α2 gene expression and poor prognosis and drug resistance in subjects with glioblastoma multiforme. J. Neuro-Oncol..

[25] Joshi B.H., Puri R.A., Leland P., Varricchio F., Gupta G., Kocak M., Gilbertson R.J., Puri R.K. (2008). Identification of interleukin-13 receptor α2 chain overexpression in situ in high-grade diffusely infiltrative pediatric brainstem glioma. Neuro-Oncology.

[26] Okada H., Low K.L., Kohanbash G., Mcdonald H.A., Hamilton R.L., Pollack I.F. (2008). Expression of glioma-associated antigens in pediatric brain stem and non-brain stem gliomas. J. Neuro-Oncol..

[27] Berlow N.E., Svalina M.N., Quist M.J., Settelmeyer T.P., Zherebitskiy V., Kogiso M., Qi L., Du Y., Hawkins C.E., Hulleman E. (2018). Il-13 receptors as possible therapeutic targets in diffuse intrinsic pontine glioma. PLoS ONE.

[28] Jaén M., Martín-Regalado Á., Bartolomé R.A., Robles J., Casal J.I. (2022). Interleukin 13 receptor alpha 2 (il13rα2): Expression, signaling pathways and therapeutic applications in cancer. Biochim. Et Biophys. Acta (BBA)-Rev. Cancer.

[29] Chen L.H., Pan C., Diplas B.H., Xu C., Hansen L.J., Wu Y., Chen X., Geng Y., Sun T., Sun Y. (2020). The integrated genomic and epigenomic landscape of brainstem glioma. Nat. Commun..

[30] Anders S., Huber W. (2010). Differential expression analysis for sequence count data. Genome Biol..

[31] Gene Ontology Consortium (2015). Gene ontology consortium: Going forward. Nucleic Acids Res..

[32] Hanzelmann S., Castelo R., Guinney J. (2013). Gsva: Gene set variation analysis for microarray and rna-seq data. Bmc Bioinformatics.

[33] Louis D.N., Perry A., Wesseling P., Brat D.J., Cree I.A., Figarella-Branger D., Hawkins C., Ng H.K., Pfister S.M., Reifenberger G. (2021). The 2021 who classification of tumors of the central nervous system: A summary. Neuro-Oncology.

[34] Louis D.N., Ohgaki H., Wiestler O.D., Cavenee W.K. (2016). WHO Classification of Tumours of the Central Nervous System.

[35] Pawlowski K.D., Duffy J.T., Tiwari A., Zannikou M., Balyasnikova I.V. (2023). Bi-specific killer cell engager enhances nk cell activity against interleukin-13 receptor alpha-2 positive gliomas. Cells.

[36] Yin Y., Rodriguez J.L., Li N., Thokala R., Nasrallah M.P., Hu L., Zhang L., Zhang J.V., Logun M.T., Kainth D. (2022). Locally secreted bites complement car t cells by enhancing killing of antigen heterogeneous solid tumors. Mol. Ther..

[37] Pandya H., Gibo D.M., Garg S., Kridel S., Debinski W. (2012). An interleukin 13 receptor α 2–specific peptide homes to human glioblastoma multiforme xenografts. Neuro-Oncology.

[38] Balyasnikova I.V., Wainwright D.A., Solomaha E., Lee G., Han Y., Thaci B., Lesniak M.S. (2012). Characterization and immunotherapeutic implications for a novel antibody targeting interleukin (il)-13 receptor α2. J. Biol. Chem..

[39] Shimato S., Natsume A., Wakabayashi T., Tsujimura K., Nakahara N., Ishii J., Ito M., Akatsuka Y., Kuzushima K., Yoshida J. (2008). Identification of a human leukocyte antigen-a24–restricted t-cell epitope derived from interleukin-13 receptor α2 chain, a glioma-associated antigen. J. Neurosurg..

[40] Yan X., Su Z., Zhang J., Wu Z., Ye S., Lu X., Wu J., Zeng Y., Zheng W. (2010). Killing effect of interleukin-13 receptor alpha 2 (il-13ralpha2) sensitized dc-ctl cells on human glioblastoma u251 cells. Cell. Immunol..

[41] Iwami K., Shimato S., Ohno M., Okada H., Nakahara N., Sato Y., Yoshida J., Suzuki S., Nishikawa H., Shiku H. (2012). Peptide-pulsed dendritic cell vaccination targeting interleukin-13 receptor α2 chain in recurrent malignant glioma patients with hla-a*24/a*02 allele. Cytotherapy.

[42] Okada H., Butterfield L.H., Hamilton R.L., Hoji A., Sakaki M., Ahn B.J., Kohanbash G., Drappatz J., Engh J., Amankulor N. (2015). Induction of robust type-i cd8+ t-cell responses in who grade 2 low-grade glioma patients receiving peptide-based vaccines in combination with poly-iclc. Clin. Cancer Res..

[43] Okada H., Kalinski P., Ueda R., Hoji A., Kohanbash G., Donegan T.E., Mintz A.H., Engh J.A., Bartlett D.L., Brown C.K. (2011). Induction of cd8+ t-cell responses against novel glioma–associated antigen peptides and clinical activity by vaccinations with α-type 1 polarized dendritic cells and polyinosinic-polycytidylic acid stabilized by lysine and carboxymethylcellulose in patients with recurrent malignant glioma. J. Clin. Oncol..

[44] Debinski W., Obiri N.I., Powers S.K., Pastan I., Puri R.K. (1995). Human glioma cells overexpress receptors for interleukin 13 and are extremely sensitive to a novel chimeric protein composed of interleukin 13 and pseudomonas exotoxin. Clin. Cancer Res..

[45] Rustamzadeh E., Vallera D.A., Todhunter D.A., Low W.C., Panoskaltsis-Mortari A., Hall W.A. (2006). Immunotoxin pharmacokinetics: A comparison of the anti-glioblastoma bi-specific fusion protein (dtat13) to dtat and dtil13. J. Neuro-Oncol..

[46] Kioi M., Seetharam S., Puri R.K. (2008). Targeting il-13rα2-positive cancer with a novel recombinant immunotoxin composed of a single-chain antibody and mutatedpseudomonas exotoxin. Mol. Cancer Ther..

[47] Candolfi M., Xiong W., Yagiz K., Liu C., Muhammad A.K., Puntel M., Foulad D., Zadmehr A., Ahlzadeh G.E., Kroeger K.M. (2010). Gene therapy-mediated delivery of targeted cytotoxins for glioma therapeutics. Proc. Natl. Acad. Sci. USA.

[48] Rossmeisl J.H., Herpai D., Quigley M., Cecere T.E., Robertson J.L., D’Agostino R.B., Hinckley J., Tatter S.B., Dickinson P.J., Debinski W. (2021). Phase i trial of convection-enhanced delivery of il13ra2 and epha2 receptor targeted cytotoxins in dogs with spontaneous intracranial gliomas. Neuro-Oncology.

[49] Ulasov I.V., Tyler M.A., Han Y., Glasgow J.N., Lesniak M.S. (2007). Novel recombinant adenoviral vector that targets the interleukin-13 receptor alpha2 chain permits effective gene transfer to malignant glioma. Hum. Gene Ther..

[50] Ou W., Marino M.P., Suzuki A., Joshi B., Husain S.R., Maisner A., Galanis E., Puri R.K., Reiser J. (2012). Specific targeting of human interleukin (il)-13 receptor α2-positive cells with lentiviral vectors displaying il-13. Hum. Gene Ther. Methods.

[51] Allen C., Paraskevakou G., Iankov I., Giannini C., Schroeder M., Sarkaria J., Puri R.K., Russell S.J., Galanis E. (2008). Interleukin-13 displaying retargeted oncolytic measles virus strains have significant activity against gliomas with improved specificity. Mol. Ther..

[52] Madhankumar A.B., Slagle-Webb B., Mintz A., Sheehan J.M., Connor J.R. (2006). Interleukin-13 receptor-targeted nanovesicles are a potential therapy for glioblastoma multiforme. Mol. Cancer Ther..

[53] Madhankumar A.B., Slagle-Webb B., Wang X., Yang Q.X., Antonetti D.A., Miller P.A., Sheehan J.M., Connor J.R. (2009). Efficacy of interleukin-13 receptor-targeted liposomal doxorubicin in the intracranial brain tumor model. Mol. Cancer Ther..

[54] Rozhkova E.A., Ulasov I., Lai B., Dimitrijevic N.M., Lesniak M.S., Rajh T. (2009). A high-performance nanobio photocatalyst for targeted brain cancer therapy. Nano Lett..

[55] Kim D., Rozhkova E.A., Ulasov I.V., Bader S.D., Rajh T., Lesniak M.S., Novosad V. (2010). Biofunctionalized magnetic-vortex microdiscs for targeted cancer-cell destruction. Nat. Mater..

[56] Bartolomé R.A., Martín-Regalado Á., Jaén M., Zannikou M., Zhang P., de Los Ríos V., Balyasnikova I.V., Casal J.I. (2020). Protein tyrosine phosphatase-1b inhibition disrupts il13rα2-promoted invasion and metastasis in cancer cells. Cancers.

[57] Vora P., Venugopal C., Salim S.K., Tatari N., Bakhshinyan D., Singh M., Seyfrid M., Upreti D., Rentas S., Wong N. (2020). The rational development of cd133-targeting immunotherapies for glioblastoma. Cell Stem Cell.

[58] Bukur J., Jasinski S., Seliger B. (2012). The role of classical and non-classical hla class i antigens in human tumors. Semin. Cancer Biol..

[59] Montemurro N., Pahwa B., Tayal A., Shukla A., De Jesus Encarnacion M., Ramirez I., Nurmukhametov R., Chavda V., De Carlo A. (2023). Macrophages in recurrent glioblastoma as a prognostic factor in the synergistic system of the tumor microenvironment. Neurol. Int..

[60] Lv Y., Zhang S., Liu Z., Tian Y., Liang N., Zhang J. (2019). Prognostic value of preoperative neutrophil to lymphocyte ratio is superior to systemic immune inflammation index for survival in patients with glioblastoma. Clin. Neurol. Neurosurg..

[61] Lin G.L., Nagaraja S., Filbin M.G., Suvà M.L., Vogel H., Monje M. (2018). Non-inflammatory tumor microenvironment of diffuse intrinsic pontine glioma. Acta Neuropathol. Commun..

[62] Chheda Z.S., Kohanbash G., Okada K., Jahan N., Sidney J., Pecoraro M., Yang X., Carrera D.A., Downey K.M., Shrivastav S. (2018). Novel and shared neoantigen derived from histone 3 variant h3.3k27m mutation for glioma t cell therapy. J. Exp. Med..

[63] Mueller S., Taitt J.M., Villanueva-Meyer J.E., Bonner E.R., Nejo T., Lulla R.R., Goldman S., Banerjee A., Chi S.N., Whipple N.S. (2020). Mass cytometry detects h3.3k27m-specific vaccine responses in diffuse midline glioma. J. Clin. Investig..

