# Supplementary material for: Expression of Interleukin-13 Receptor Alpha 2 in Brainstem Gliomas
Source: Cancers (Basel). 2024 Jan 3;16(1):228. doi: 10.3390/cancers16010228 (PMC10777982; doi:10.3390/cancers16010228)
Supplement: Supplementary file 1 [file cancers-16-00228-s001.zip › Supplementary Figures.pdf]

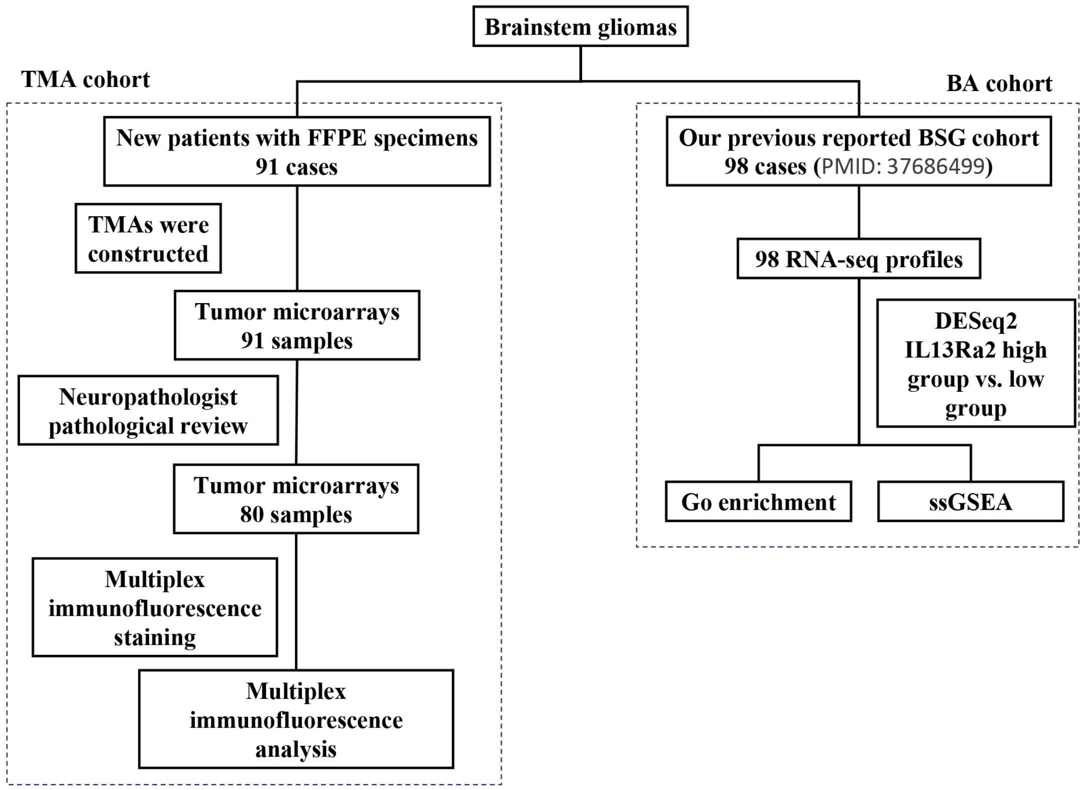

Figure S1 The workflow of this study

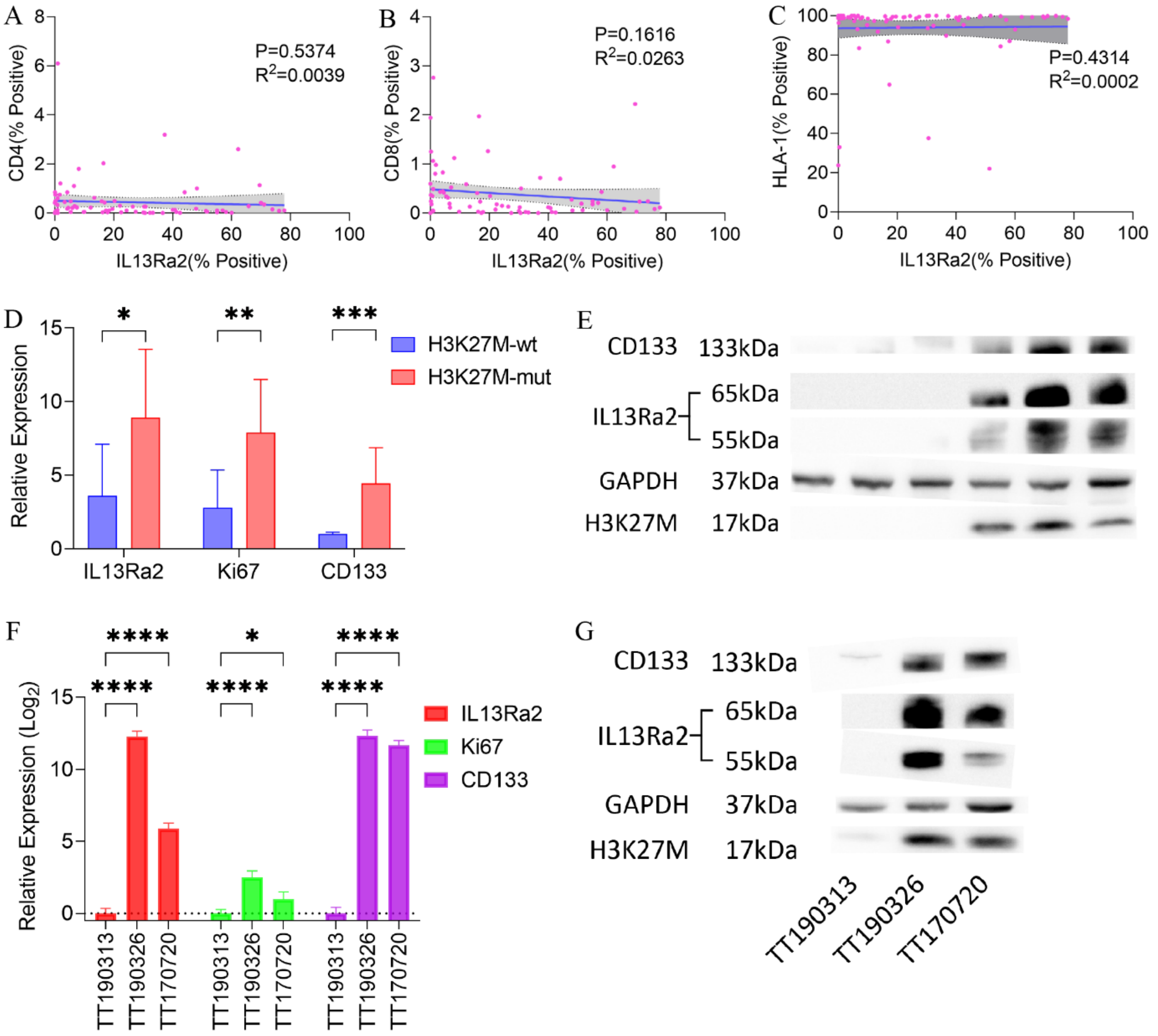

Figure S2 Co-staining results in the TMA cohort & Western blot and qPCR results of BSG tumor tissues and cells. Linear regression results of IL13Ra2 staining extent and CD4(A), CD8(B), HLA1(C) staining extent in TMA queue; qPCR(D) and Western blot (E) results of BSG tumor tissues; qPCR(F) and Western blot (G) results of BSG cells.
