# Supplementary material for: Expression of Interleukin-13 Receptor Alpha 2 in Brainstem Gliomas
Source: Cancers (Basel). 2024 Jan 3;16(1):228. doi: 10.3390/cancers16010228 (PMC10777982; doi:10.3390/cancers16010228)
Supplement: Supplementary file 1 [file cancers-16-00228-s001.zip › Supplementary Tables.pdf]

Table S1 Clinical characteristics of TMA cohort and BA cohort

		TMA cohort	BA cohort
Total		80	98
Gender	Female	43	48
	Male	37	50
Age	≤14 years	41	38
	> 14 years	39	60
Location	Thalamus	5	0
	Midbrain	14	14
	Pons	38	54
	Medulla oblongata	23	30
WHO-Grade	1	9	8
	2	19	23
	3	3	9
	4	49	60
DIPG	No	44	68
	Yes	36	31

Table S2 IL13Ra2 expression in different BSG subgroups in TMA cohort

		IL13Ra2									
		Total	≤10%	＞10%	P-value	≤20%	＞20%	P-value	≤50%	>50%	P-value
Total		80	33	47		44	36		65		
Gender	Female	43	16	27	0.429	22	21	0.457	32	11	0.091
	Male	37	17	20		22	15		33	4	
Age	≤14 years	41	17	24	0.968	24	17	0.514	34	7	0.694
	＞14 years	39	16	23		20	19		31	8	
Location	Thalamus	5	2	3	0.008	2	3	0.066	4	1	0.792
	Midbrain	14	10	4		11	3		12	2	
	Pons	38	9	29		16	22		29	9	
	Medulla oblongata	23	12	11		15	8		20	3	
WHO-Grade	1	9	8	1	<0.0001	9	0	<0.0001	9	0	0.007
	2	19	15	4		18	1		19	0	
	3	3	1	2		2	1		3	0	
	4	49	9	40		15	34		34	15	
DIPG	No	44	25	19	0.002	29	15	0.030	37	7	0.472
	Yes	36	8	28		15	21		28	8	
H3F3A	Wild-type	14	14	0	<0.0001	14	0	<0.0001	14	0	0.070
	Mutant	22	0	22		4	18		17	5	
	Unknown	44	19	25		26	18		34	10	
H3.3	(H3.3-mutant) ≤10%	33	28	5	<0.0001	31	2	<0.0001	33	0	<0.001
	(H3.3-wild) ＞10%	47	5	42		13	34		32	15	
H3.3	(median)	43.37%	0.10%	57.91%	<0.0001	0.41%	62.62%	<0.0001	8.66%	66.11%	<0.001
Ki67	(median)	10.60%	1.11%	25.29%	<0.0001	1.85%	29.43%	<0.0001	4.69%	64.61%	<0.0001
CD133	(median)	1.34%	0.04%	4.51%	<0.0001	0.08%	9.72%	<0.0001	0.69%	46.66%	<0.0001
HLA1	(median)	99.19%	99.22%	98.96%	0.126	99.01%	99.20%	0.832	99.03%	99.30%	0.848
CD4	(median)	0.16%	0.22%	0.10%	0.183	0.22%	0.10%	0.134	0.18%	0.10%	0.661
CD8	(median)	0.20%	0.35%	0.13%	0.018	0.35%	0.12%	0.013	0.22%	0.19%	0.943

Table S3 Multiple linear regression of IL13Ra2 expression in TMA cohort

Variables	β	95% CI		*P* value
Gender	-2.186	(-10.323	5.95)	0.594
Age	0.015	(-0.257	0.287)	0.912
Location	2.044	(-2.664	6.753)	0.390
WHO-Grade	5.248	(-1.005	11.502)	0.099
DIPG	-3.409	(-14.226	7.409)	0.532
H3.3-mutant	24.451	(12.024	36.877)	<0.001

Table S4 Spearman correlation analyses of IL13Ra2 expression in TMA cohort

		H3.3K27M	Ki67	CD133	HLA1	CD4	CD8
IL13Ra2	correlation coefficient	0.796	0.719	0.826	-0.089	-0.070	-0.193
	P-value	<0.0001	<0.0001	<0.0001	0.431	0.537	0.094
	Number of cases	80	80	80	80	80	76*

* FFPE tumor tissue sections for four cases have been exhausted.

Table S5 Multivariate Cox regression for survival analysis in TMA cohort

TMA cohort	Univariate Cox regression		Multivariate Cox regression	
	HR (95% CI)	P	HR (95% CI)	P
Gender	1.408(0.704-2.815)	0.334	0.887(0.425-1.851)	0.749
Age	0.981(0.958-1.005)	0.126	0.992(0.964-1.020)	0.563
DIPG	3.383(1.633-7.009)	0.001	2.061(0.818-5.194)	0.125
IL13Ra2(>20%)	2.520(1.228-5.099)	0.012	0.822(0.375-1.799)	0.623
H3F3A (H3.3>10%)	9.585(3.268-28.106)	<0.0001	9.228(2.685-31.714)	<0.0001
Ki67	1.019(1.007-1.031)	0.001	1.003(0.988-1.017)	0.725
CD133	1.014(0.996-1.033)	0.132		
HLA-1	1.005(0.984-1.026)	0.668		
CD4	0.447(0.162-1.232)	0.120		
Grade (IV)		0.006		
I	<0.001	0.978		
II	0.019(0.002-0.173)	<0.0001		
III	0.098(0.011-0.902)	0.040		

Table S6 Multivariate Cox regression for survival analysis in BA cohort

BA cohort	Univariate Cox regression		Multivariate Cox regression	
	HR	P	coef	P
Age	0.978	0.015	0.976	0.150
Gender	0.989	0.966	-	-
Location (Midbrain)				
Medulla	0.997	0.995	1.594	0.489
Pons	2.982	0.022	1.985	0.336
DIPG	3.394	<0.001	1.039	0.936
Grade (IV)	-	-	-	-
I	<0.001	0.997	<0.001	0.998
II	0.076	<0.001	0.206	0.001
III	0.276	<0.001	0.573	0.158
H3F3A	8.0172	<0.001	1.157	0.788
Methylation Cluster(H3-Pon)	-	-	-	-
IDH	0.049	<0.001	0.075	0.009
H3-Medulla	0.268	<0.001	0.599	0.380
PA-like	<0.001	0.996	<0.001	0.996
IL13RA2_Group	2.128	0.009	1.742	0.144
